# Dementia burden among women in China: A cross-national comparative analysis with the United States and Japan using GBD 2021

**DOI:** 10.1097/MD.0000000000047361

**Published:** 2026-01-23

**Authors:** Ziyi Li, Wenjun Xiu, Jiefang Duan, Yu Zhang, Xiaobei Dou, Jie Zhang, Guangxin Sheng, Yanli Liu

**Affiliations:** a School of Nursing, Shandong University of Traditional Chinese Medicine, Jinan, Shandong, China.

**Keywords:** China, dementia, disease burden, Japan, USA, women

## Abstract

This study aims to quantify the burden of Alzheimer’s disease and other dementias among women in China from 1990 to 2021 and compare these patterns with those in the United States and Japan, identifying drivers and informing targeted prevention and intervention strategies. We extracted dementia data for women aged ≥ 40 years in China, the United States, and Japan from the GBD 2021 database, including prevalence, mortality, and disability-adjusted life years (DALYs). Trends in age-standardized prevalence rate, age-standardized death rate, and age-standardized DALY rate from 1990 to 2021 were summarized using estimated annual percentage change (EAPC) and percent change. Factor decomposition quantified the contributions of population aging, population growth, and epidemiological change to female dementia mortality. Pearson correlation examined associations between age-standardized DALY rates and the socio-demographic index. A Bayesian age-period-cohort model projected dementia prevalence and mortality among Chinese women from 2022 to 2036. In 2021, China had 10.83 million women living with dementia and 3,28,400 dementia-related deaths. The age-standardized prevalence rate, age-standardized death rates, and age-standardized DALY rate among Chinese women were 1025.11, 33.80, and 631.38 per 1,00,000, respectively – each higher than the corresponding rates in the United States (855.19, 31.60, and 563.46 per 1,00,000) and Japan (759.99, 29.57, and 516.08 per 1,00,000). Across all 3 countries, the dementia burden among women exceeded that among men. In China, prevalence peaked at ages 75 to 79. Elevated fasting plasma glucose and high body mass index were the principal risk factors. Population growth and aging were the main drivers of rising dementia mortality among Chinese women. Projections indicate that both prevalence and mortality among Chinese women will continue to increase through 2036. The burden of dementia among women in China exceeds that in the United States and Japan and is projected to grow further. Strengthening early detection, midlife health management, and health insurance coverage, with particular attention to women’s health needs, is recommended to mitigate the future burden.

## 1. Introduction

With global population aging, dementia has emerged as a major public health challenge.^[1]^ Alzheimer’s disease is a progressive neurodegenerative disorder characterized by gradual cognitive deterioration and is the most common cause of dementia.^[2]^ According to data from the 2021 Alzheimer’s Association Report, a new case of dementia is diagnosed every 3 seconds worldwide^[3]^; by 2050, the global number of dementia patients is projected to rise from the current 55 million to 139 million.^[4]^ Dementia not only leads to functional impairment and a significant decline in quality of life for patients but also imposes a heavy care and economic burden on families and society.^[5]^

Gender differences are particularly pronounced in the epidemiology of dementia and in the utilization of health resources. Numerous studies report that dementia prevalence and incidence are significantly higher in women than in men,^[6]^ potentially reflecting factors such as women’s greater longevity, postmenopausal changes in estrogen, and gendered social roles.^[7]^ This disparity is especially marked in China: in 2019 female life expectancy in mainland China was 81.0 years versus 74.8 years for males, and projections suggest women will outlive men by approximately 7 years by 2035, thereby extending their period of dementia risk.^[8]^ Moreover, female patients incur substantially higher costs and place greater demands on diagnostic, treatment, social support, and long-term care resources than male patients.^[9]^ Thus, analyzing the burden of dementia from a sex-specific perspective is essential for promoting health equity and for optimizing the allocation of eldercare resources.^[10,11]^

Recent studies have advanced our understanding of dementia epidemiology, risk factors, and care-cost estimation.^[12–14]^ Although age-standardized incidence rates have declined in many high-income countries,^[15]^ they remain on the rise in several middle-income settings, including China.^[16]^ Cross-national comparisons have largely emphasized Western countries, with limited attention to comparability across Asian contexts – particularly China and Japan – in terms of cultural traditions and family structures.^[17]^ Dementia imposes substantial socioeconomic burdens worldwide, and China, the United States, and Japan are projected to bear the largest economic impacts.^[18]^ China faces a large and regionally uneven aging population^[19]^; the United States has relatively well-developed health and long-term care systems but contends with high costs^[20]^; and Japan has developed distinctive policies in response to its super-aged society and entrenched family caregiving traditions.^[21,22]^ Despite these institutional differences, cross-cultural comparative research from a gender perspective is scarce, potentially limiting policymakers’ ability to accurately identify the specific needs of women living with dementia and to design precise, effective prevention and care strategies targeting women.

Using data from the 2021 Global Burden of Disease (GBD) study, this paper examines the burden of Alzheimer’s disease and other dementias in China from a female perspective and compares it with that in the United States and Japan. By analyzing cross-national epidemiological indicators, we aim to provide evidence to inform targeted prevention and intervention strategies.

## 2. Materials and methods

### 2.1. Data sources

Dementia data were obtained from the GBD 2021 study.^[23]^ GBD 2021 estimated the burden of 371 diseases and injuries and 88 risk factors across 204 countries and territories. Data were extracted using the following parameters: location (China, United States, Japan), sex (female), age (≥40 years), years (1990–2021), and metrics (prevalence, deaths, DALYs, age-standardized rates). Comprehensive information on data sources and related metadata is available through the online data platform at: http://ghdx.healthdata.org/gbd-results-tool.

Ethics statement: Data used in this study were obtained from the publicly available GBD 2021 database and contain no individual-level identifiable information.

### 2.2. Statistical analysis

The GBD world standard population age structure was used to calculate age-standardized prevalence rates (ASPR), age-standardized death rates (ASDR), and age-standardized DALY rates. Temporal trends in age-standardized rates from 1990 to 2021 were assessed by estimating the annual percentage change (EAPC) with corresponding 95% uncertainty interval (UI).The EAPC is computed as 100 × [exp(β) − 1],^[24]^ reflecting the annual percentage change. A linear regression model was utilized to derive the 95% confidence interval (CI) for the EAPC. An increasing trend is defined when both the EAPC and the lower CI bound are positive; a decreasing trend is indicated if both the EAPC and the upper CI bound are negative.

### 2.3. Research methods

Risk factors for dementia in women – including high body mass index (BMI), elevated fasting plasma glucose, and smoking – were examined to assess their impact across different years and age groups.

The Das Gupta method was used for decomposition analysis, which assumes a linear relationship between age structure, population growth, and epidemiological factors, isolating the independent contribution of each factor while holding the others constant.^[25]^ This method accounts for interactions among these factors and provides a comprehensive decomposition of temporal changes in the disease burden. We calculated the expected change due to population growth, the additional change due to population aging, and attributed the remaining change to epidemiological factors.^[26]^

A Bayesian age-period-cohort (BAPC) model was used to project the ASPR and ASDR of dementia among Chinese women from 2022 to 2036. The BAPC model was specified as: log(λ_*ij*_) = µ + α_*i*_ + β_*j*_ + γ_*k*_ + ε,where μ is the intercept, ε is the random error term, and α_*i*_, β_*j*_, and γ_*k*_ represent the age, period, and cohort effects, respectively.^[27]^Independent second-order random walk (RW2) priors were assigned to the age, period, and cohort effects. Model fitting was performed using the BAPC package via Integrated Nested Laplace Approximation. Model fit and convergence were assessed by inspecting posterior distributions and the deviance information criterion (DIC).

To quantify uncertainty in the projections, 95% confidence interval (CI) were calculated from the posterior distributions. Specifically, for each projected value, the 2.5th and 97.5th percentiles of the posterior samples were taken as the lower and upper bounds of the CI, respectively, reflecting uncertainty arising from the age, period, and cohort effects as well as residual variation.

The analyses were performed using R version 4.2.2 (Vienna, Austria), RStudio (Boston), Microsoft Excel 2022 (Redmond) and data visualizations were created using the ggplot2 package.

## 3. Results

### 3.1. Current burden of dementia among women in China, the United States, and Japan

In 2021, the number of women living with dementia in China and the number of dementia-related deaths were 1,08,28,600 and 3,28,400, respectively. During the same period, the ASPR, ASDR, and DALY rate for dementia among Chinese women were 1025.11 (95% UI: 879.04–1186.81), 33.80 (95% UI: 8.6–87.19), and 631.38 (95% UI: 305.95–1318.24) per 1,00,000, respectively – all higher than the corresponding rates in the United States (855.19 [95% UI: 742.39–975.4], 31.60 [95% UI: 8.49–78.01], and 563.46 [95% UI: 270.18–1168.59] per 1,00,000) and Japan (759.99 [95% UI: 657.37–866.96], 29.57 [95% UI: 8.61–66.49], and 516.08 [95% UI: 256.41–1001.7] per 1,00,000, respectively; Table S1, Supplemental Digital Content, https://links.lww.com/MD/R208).

Compared with 1990, the ASPR among Chinese women increased by 0.31 (EAPC: 0.46, 95% CI: 0.36–0.55), representing the largest rise among the 3 countries. The ASDR decreased by −0.02 (EAPC: −0.20, 95% CI: −0.24 to −0.17), while the age-standardized DALY rate increased by 0.06, a change that was not statistically significant (EAPC: −0.02%, 95% CI: −0.06 to 0.02). Overall, in 2021 the dementia burden among women in China exceeded that in the United States and Japan, and in all 3 countries the burden among women was higher than that among men.

**Figure 1.**
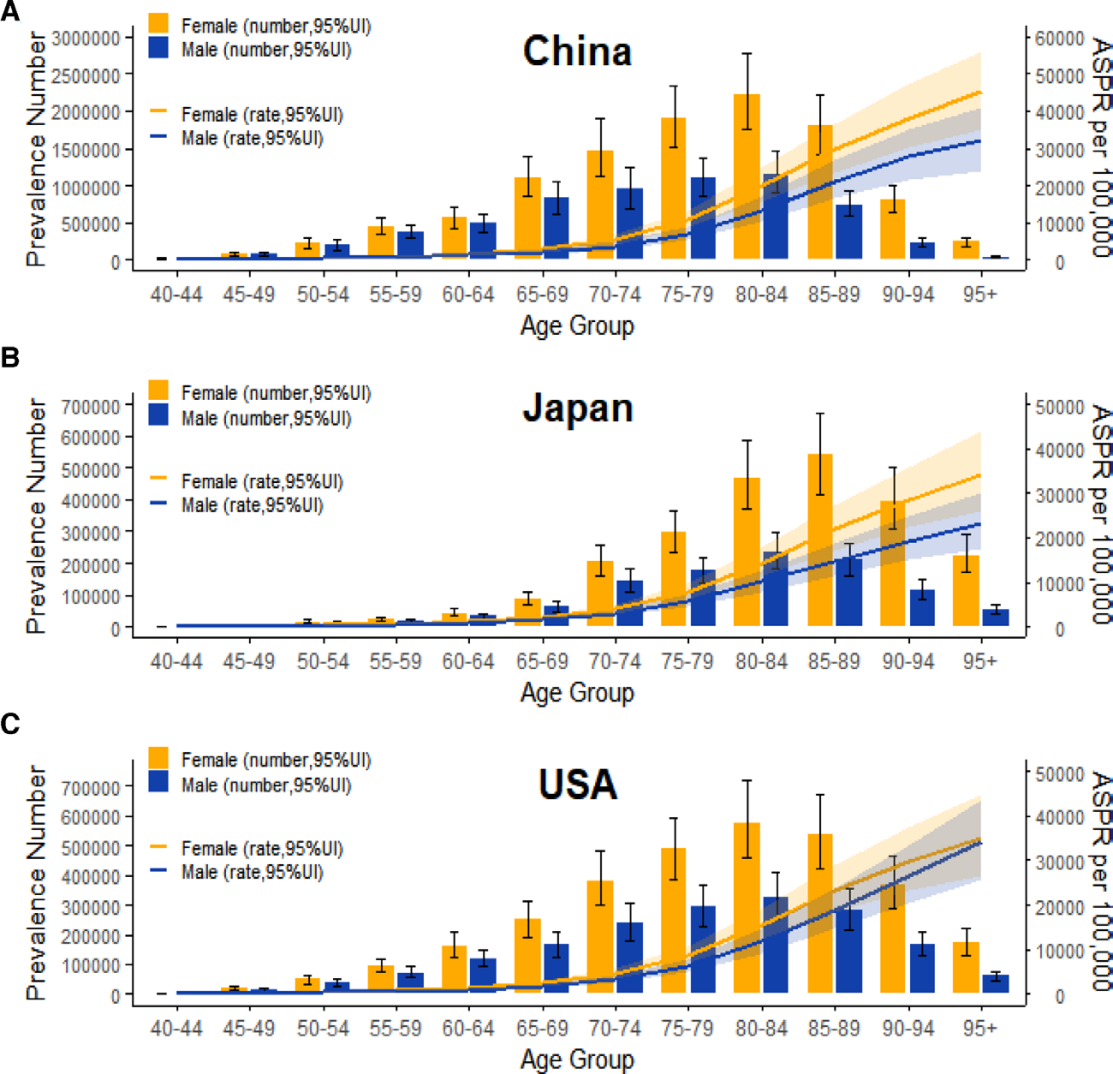
Dementia prevalence by age group in women in China, the United States, and Japan in 2021.

The ASPR, ASDR, and age-standardized DALY rates for dementia among women in all 3 countries increased with advancing age(Figs S1 and S2, Supplemental Digital Content, https://links.lww.com/MD/R208). In terms of prevalent case numbers, the peak prevalence occurred at ages 80 to 84 in both China and the United States, whereas Japan’s peak was reached later, at ages 85 to 89 (Fig. 1).

### 3.2. Risk factors for dementia among women in China, the United States, and Japan

**Figure 2.**
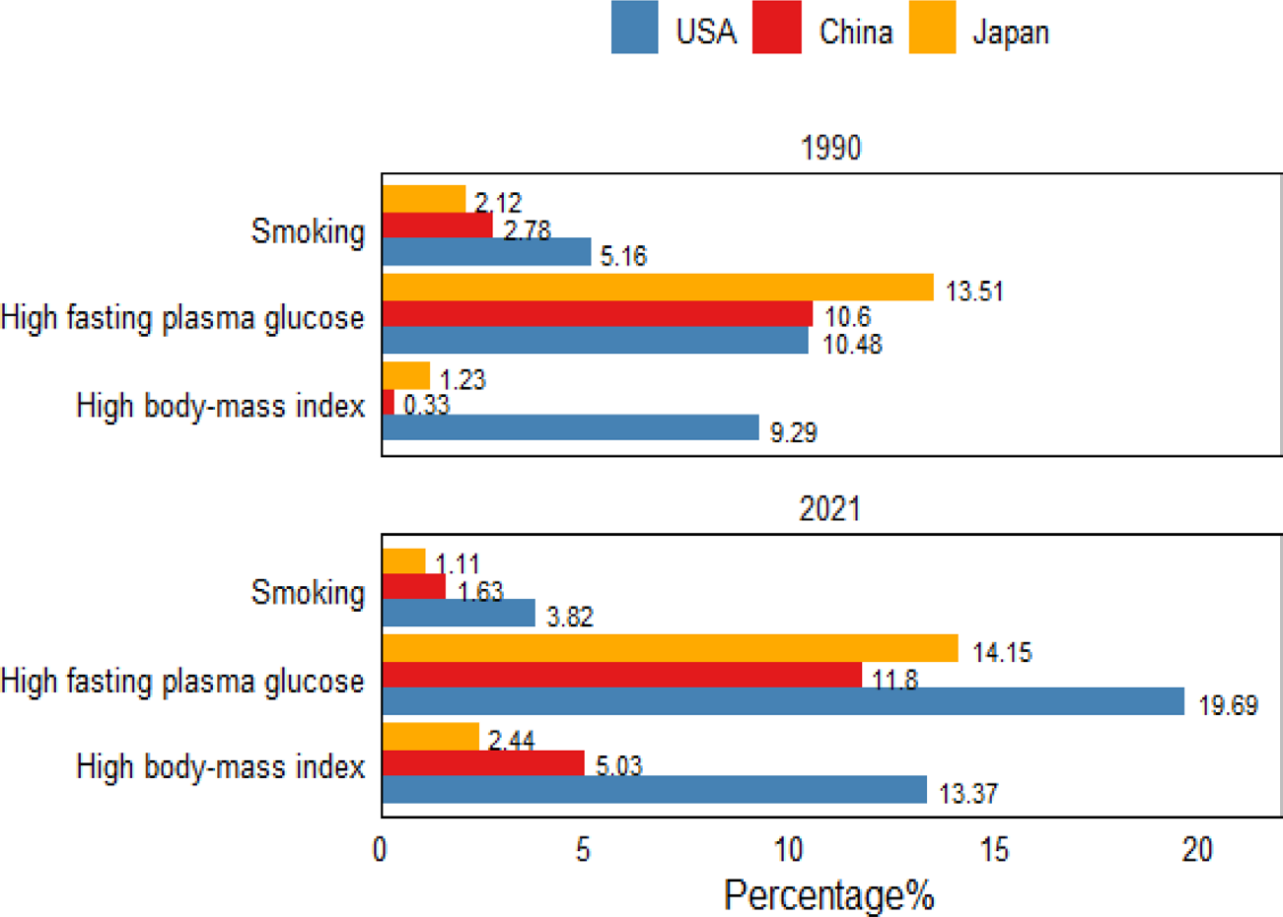
Risk factor analysis of dementia attributed to DALYs in women in China, the United States, and Japan in 1990 and 2021. DALY = disability-adjusted life year.

**Figure 3.**
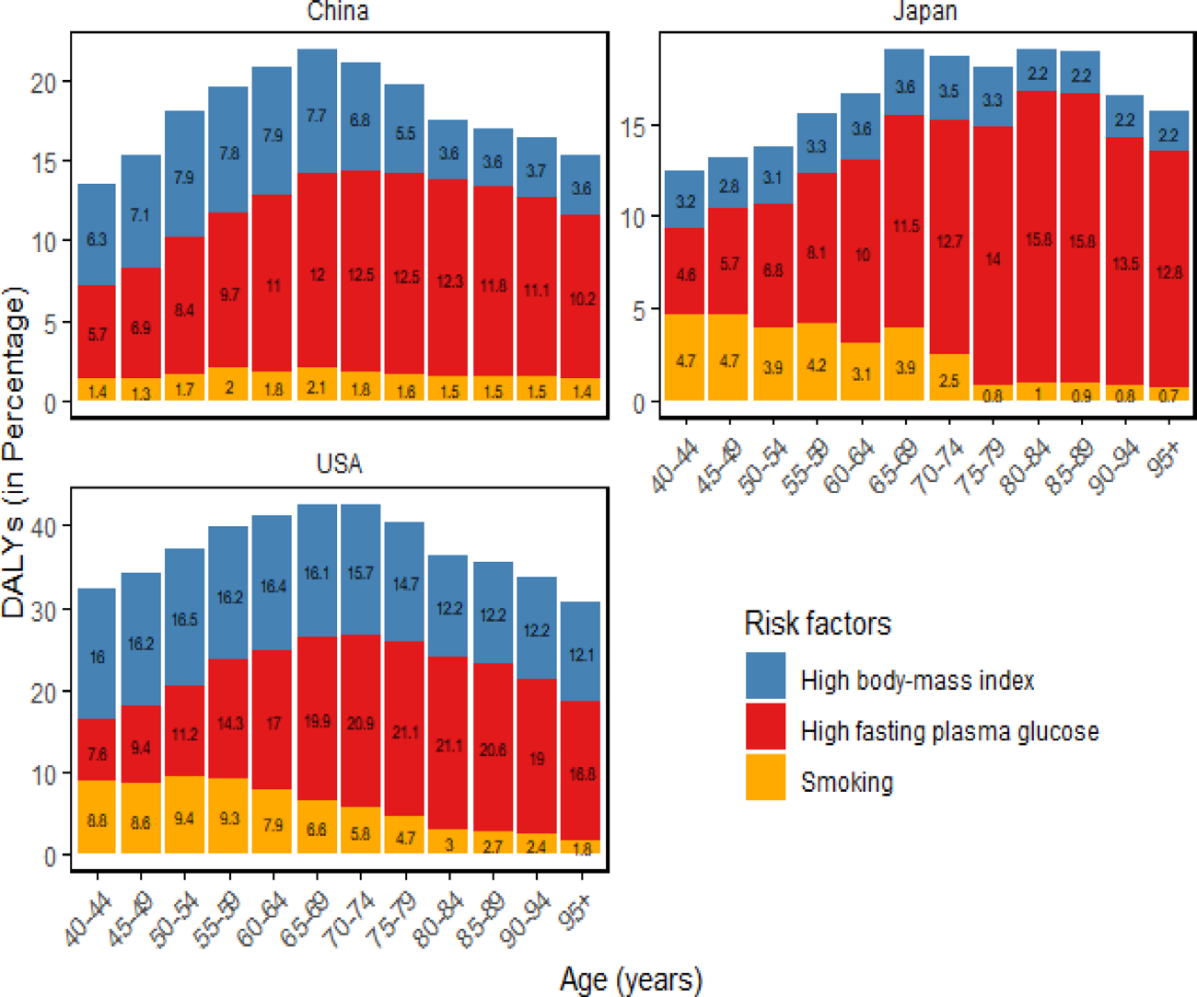
Risk factor analysis of dementia attributed to DALYs by age group in Women in China, the United States, and Japan in 2021. DALY = disability-adjusted life year.

Figs 2 and 3 together illustrate both the national shifts and the age-specific patterns in dementia risk attributable to the 3 major factors between 1990 and 2021. At the national level, high fasting plasma glucose remained the dominant contributor across all 3 countries – its share rose by 1.2% in China and was highest in the United States – while high BMI climbed in importance in China, moving from the third to the second leading risk by 2021. Between 1990 and 2021 the proportion of DALYs among Chinese women attributable to high BMI increased by 4.7% (with similar upward trends in the United States [4.08%] and Japan [0.32%]), whereas the burden due to smoking declined in all 3 settings, accounting for only 1.11% of DALYs in China in 2021. Age-stratified analyses show that these national rankings largely persist across age groups: the combined impact of the 3 risks rises through midlife, peaks at ages 65 to 69, and then falls; the smallest relative impact is seen in the 40 to 44 age group in China and Japan but in women aged ≥95 years in the United States.

### 3.3. Decomposition analysis of factors contributing to dementia-related mortality among women in China, the United States, and Japan

**Figure 4.**
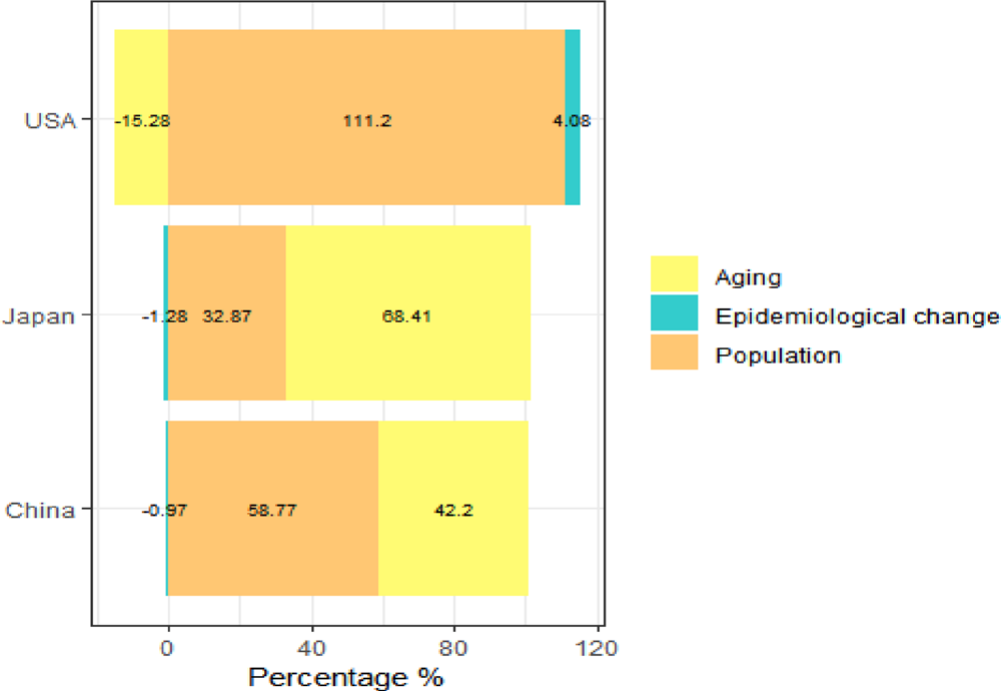
Factor decomposition analysis of dementia mortality in women in China, the United States, and Japan from 1990 to 2021.

Analysis of factors driving dementia-related deaths among women reveals that population growth and aging are the predominant contributors, with population growth exerting the greatest effect. Population growth accounted for 58.77%, 111.20%, and 32.87% of dementia-related female mortality in China, the United States, and Japan, respectively. In contrast, the impact of population aging varied across countries, contributing 42.20% in China, 68.41% in Japan, and −15.28% in the United States, indicating a more pronounced influence of aging in Japan compared to China. Epidemiological changes had a relatively minor effect on dementia-related mortality among women in China and Japan, insufficient to counterbalance the effects of population growth and aging. Conversely, in the United States, epidemiological changes modestly contributed to the increase in deaths, accounting for 4.08% (Fig. 4).

### 3.4. Correlation analysis between age-standardized DALY rates and SDI in China, the United States, and Japan

**Figure 5.**
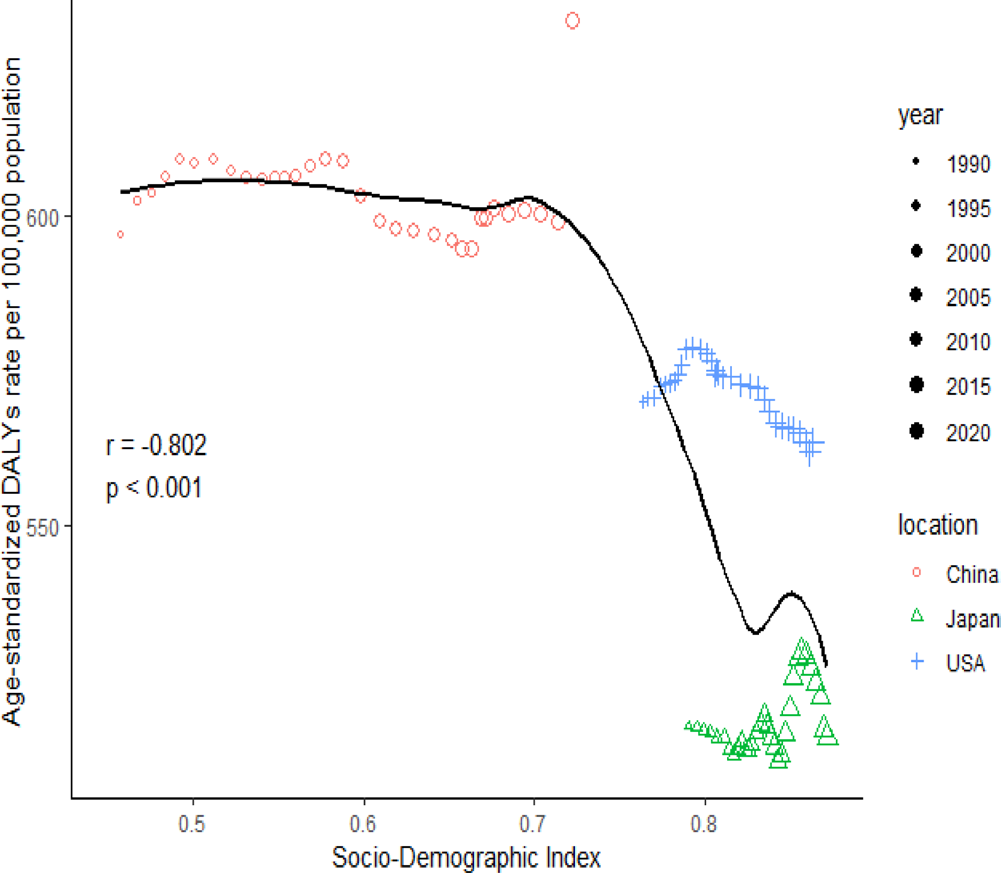
Correlation analysis of age-standardized DALY rates for dementia and SDI in women in China, the United States, and Japan. DALY = disability-adjusted life year, SDI = socio-demographic index.

Pearson correlation analysis revealed a significant negative association between overall age-standardized DALY rates and the socio-demographic index in China, the United States, and Japan from 1990 to 2021 (Fig. 5).

### 3.5. Projection of dementia prevalence and mortality trends among Chinese women, 2022–2036

**Figure 6.**
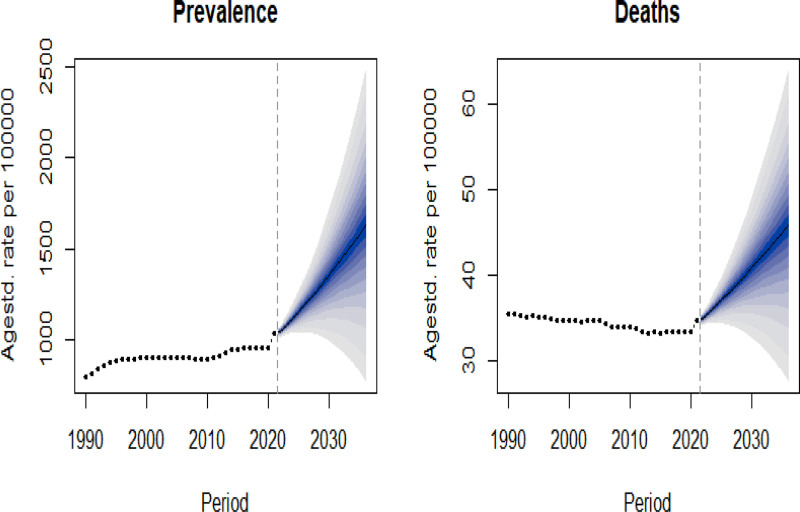
Projection of dementia prevalence and mortality trends among Chinese women, 2022–2036.

Using BAPC model, the prevalence and mortality rates of dementia among Chinese women were projected over the next 14 years. The results indicate that both the ASPR and ASDR are expected to rise from 2022 to 2036. Specifically, the ASPR is projected to increase from 1039.61 per 1,00,000 in 2021 to 1627.18 per 1,00,000, representing a 56.53% increase, while the ASDR is anticipated to grow from 34.58 to 45.76 per 1,00,000, corresponding to a 32.33% increase (Fig. 6).

## 4. Discussion

### 4.1. Integrated effects of age, epidemiology, and population structure on the dementia burden among women

This study’s findings indicate that in 2021, dementia burden was consistently higher among women than men in China, the United States, and Japan, with Chinese women experiencing a greater burden than their counterparts in the United States and Japan. Age exerted a significant influence on dementia burden, aligning with previous studies.^[28]^ Dementia prevalence among Chinese women increased with age, and similar overall patterns were observed in the United States and Japan; however, the overall dementia prevalence among women in the United States has been declining.^[29]^

A study examining risk factors for dementia in women identified high fasting blood glucose, elevated BMI, and smoking as significant contributors across China, the United States, and Japan, with high fasting glucose and BMI emerging as the primary risk factors. Compared with the United States and Japan, China has a larger increase in high fasting blood sugar and BMI, which may be related to factors such as socioeconomic development, improved living standards of women leading to changes in dietary structure and reduced physical activity.^[30]^ Furthermore, social norms impose less stringent body shape expectations on middle-aged and elderly women compared to younger cohorts, and improvements in their social status contribute to a higher prevalence of elevated BMI in this demographic,^[31]^ thereby elevating their dementia risk.

Factor decomposition analysis reveals that population growth is a common driver of increased dementia-related mortality among women in China, the United States, and Japan, with population aging exerting a greater influence in China and Japan. According to the seventh national census, China’s total population grew at an average annual rate of 0.53%, with the proportion aged 65 and older rising to 4.6% over the past decade.^[32]^ Compared to China, Japan faces a more pronounced impact from population aging, with the number of individuals aged 75 and above projected to exceed 20 million by 2025.^[33]^ Epidemiological changes have contributed to a modest reduction in dementia-related deaths among women in China and Japan, whereas the opposite trend is observed in the United States. These differences likely reflect variations in healthcare systems, economic development, and lifestyle factors across the 3 countries. The notable decline in dementia-related mortality among Chinese women may be attributable to recent healthcare improvements.

China’s high dementia burden may reflect factors beyond population aging and growth. Older generations of Chinese women typically had fewer opportunities for formal education than their counterparts in the United States and Japan. Lower educational attainment is associated with reduced cognitive reserve, which increases vulnerability to dementia later in life.^[34]^ In addition, developing or resource-limited regions – including parts of rural China – often lack adequate geriatric care, cognitive screening, and early diagnostic services. Such limitations can lead to delayed or missed diagnoses, resulting in a statistically higher recorded disease burden.^[35]^

The United States and Japan, by contrast, have more established systems for preventive screening, chronic disease management, and community-based long-term care. China is still in the process of integrating gender perspectives into national aging and dementia policies and expanding midlife metabolic interventions and cognitive screening within primary care settings, which may limit the extent to which women benefit from preventive measures.^[34]^

Finally, cross-national variations in screening coverage, healthcare-seeking behavior, and cause-of-death classification may introduce bias and affect data comparability. These methodological differences should be considered when interpreting international disparities in dementia burden.^[36]^

### 4.2. Drawing on United States and Japan’s experiences to mitigate China’s dementia burden

BAPC model projections indicate that dementia prevalence and mortality rates among Chinese women are expected to increase over the next 14 years, so targeted prevention and management strategies should be developed. Given that high BMI leads to an increase in disability-adjusted life years (DALYs), it is recommended that weight management interventions be promoted in middle-aged women, such as community-based lifestyle adjustment programs, dietary guidance, and physical exercise promotion. There is evidence that controlling metabolic risk factors in middle age can significantly reduce the risk of dementia in late life.^[37]^ According to the decomposition results, population aging is the largest contributor to the rising burden of dementia in China. This highlights the need to establish a comprehensive long-term care system and conduct early cognitive screening for the rapidly growing elderly female population.^[38]^ Japan has a well-developed community-based dementia care and early identification system, and its experience can provide valuable reference for China.

As the world’s first super-aged society, Japan has enacted the Basic Law on Dementia for the Promotion of an Inclusive Society^[39]^ and the “New Orange Plan.”^[40]^However, despite these efforts, the rapid population aging has led to a continued rise in dementia incidence among Japanese women, indicating that legislation and community support alone are insufficient to reverse overall epidemiological trends. Furthermore, although Japan’s “Women Researchers Support Program” promotes female participation in higher education and research – potentially enhancing the protective effects of education on cognitive health – its reach remains limited in rural and remote areas.^[41]^ Building on these achievements and recognizing these limitations, China should draw from Japan’s experiences in community support and policy safeguards while placing greater emphasis on risk management for perimenopausal women, educational equity, and health interventions targeting rural women to foster more gender-responsive dementia prevention and control.

Research indicates that across all birth cohorts, individuals in the United States are less likely to develop dementia at given ages compared to earlier generations.^[42]^ Mukadam et al^[15]^ attribute this trend primarily to improvements in educational attainment and cardiovascular health, driven by expanded early compulsory education policies and proactive cardiovascular risk management. The United States has implemented measures such as the Medicare Annual Wellness Visit (AWV) and the “Million Hearts” initiative,^[43,44]^ alongside payment reforms like the Comprehensive Primary Care Plus (CPC+) model.^[45]^

In China, however, the benefits of enhanced educational opportunities may not yet be fully realized, due to the relatively recent expansion of compulsory education and uneven distribution of educational resources across urban and rural areas, resulting in delayed overall educational attainment among women. Furthermore, significant gaps remain in primary and secondary prevention of cardiovascular diseases, especially among women.^[46]^ Therefore, China should prioritize women’s education, intensify targeted educational and preventive strategies, and accelerate advancements in cardiovascular risk management.

## 5. Conclusion

In conclusion, the dementia burden among women in China exceeds that of the United States and Japan. Prevalence and mortality rates are projected to continue rising over the next 14 years. Elevated fasting blood glucose and high BMI represent the primary risk factors in China, with a notable increase in dementia-related DALYs attributable to high BMI among Chinese women. Although epidemiological shifts exert some influence on mortality, population growth and aging remain the predominant drivers. Accordingly, Chinese policymakers should leverage lessons from developed countries in early screening and health management while tailoring dementia prevention and control strategies to reflect the cultural nuances and resource disparities across female populations in different regions.

## 6. Limitation

This study has several limitations. First, the epidemiological data come from GBD 2021, which only covers dementia burden through 2021. GBD estimates are modeled from source data of variable quality and may therefore contain inaccuracies or biases that could affect the robustness of our conclusions. Second, cross-country comparisons are limited by differences in data collection, case definitions, diagnostic practices and reporting systems among countries; these heterogeneities make direct comparisons difficult and increase the risk of error. Clarifying the causes of these inter-country differences would be important for improving the policy relevance and interpretability of the results.

## Acknowledgments

We thank the Global Burden of Disease (GBD) study investigators for making the GBD 2021 data publicly available.

## Author contributions

**Data curation:** Ziyi Li, Wenjun Xiu, Jie Zhang, Guangxin Sheng.

**Methodology:** Jiefang Duan, Yu Zhang, Xiaobei Dou.

**Software:** Ziyi Li.

**Writing – original draft:** Ziyi Li.

**Writing – review & editing:** Yanli Liu.

## Supplementary Material


