# Supplementary material for: Dementia burden among women in China: A cross-national comparative analysis with the United States and Japan using GBD 2021
Source: Medicine (Baltimore). 2026 Jan 23;105(4):e47361. doi: 10.1097/MD.0000000000047361 (PMC12851736; doi:10.1097/MD.0000000000047361)
Supplement: Supplementary file 1 [file medi-105-e47361-s001.docx]

**Supplementary Material 1**

A
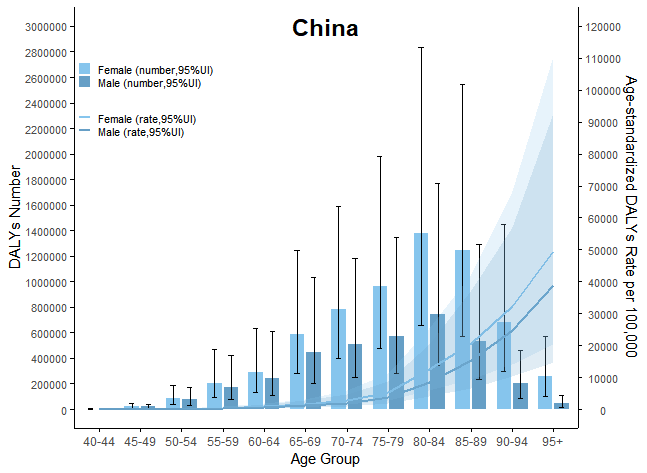
B
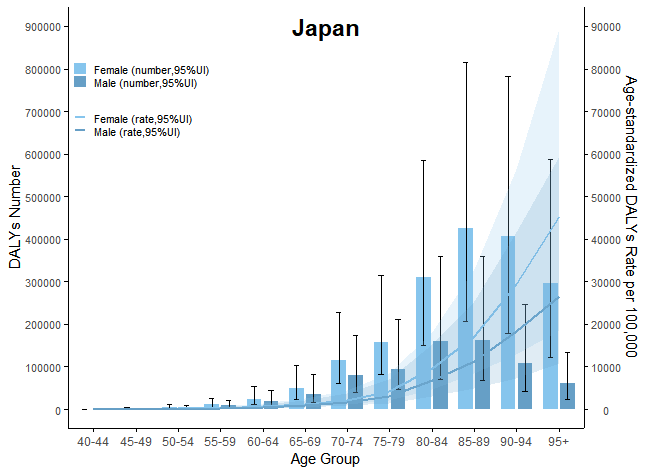
C
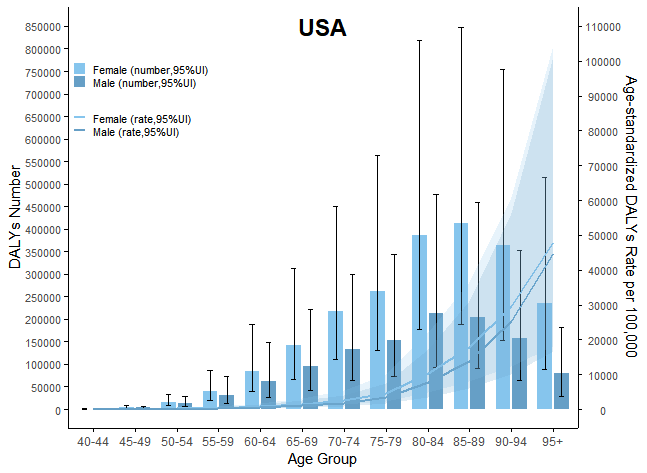
**Fig S1 Dementia DALYS by age group in women in China, the United States, and Japan in 2021**

**Supplementary Material 2**

A
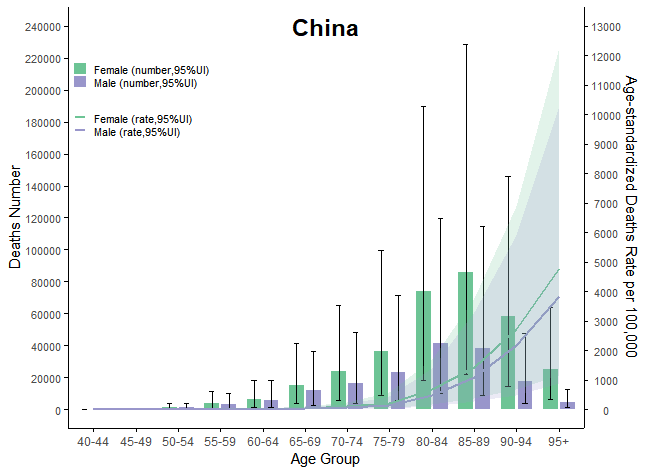
B
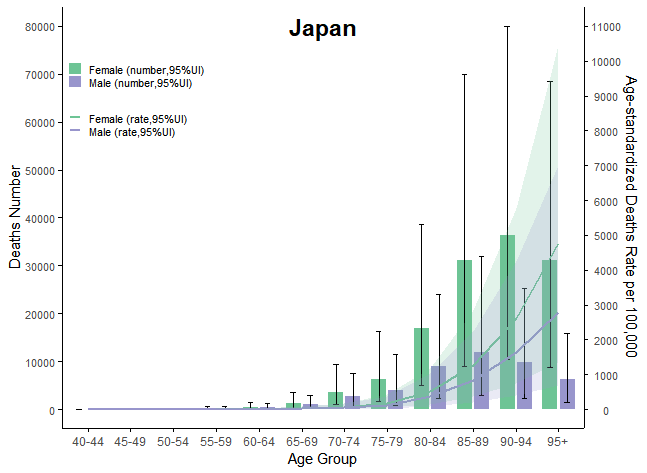
C
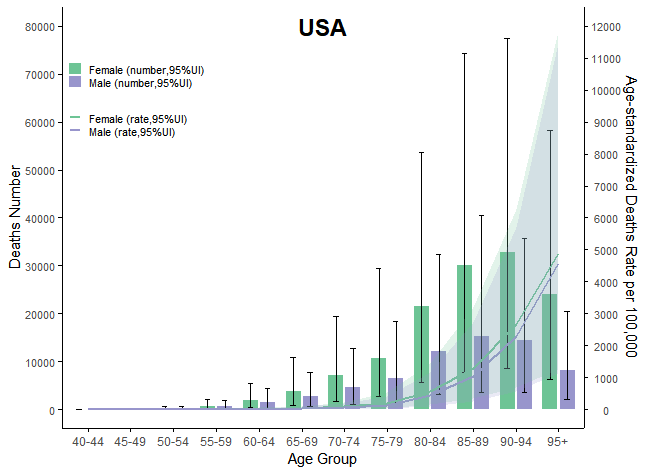
**Fig S2 Dementia Deaths by age group in women in China, the United States, and Japan in 2021**

| **Table S1 The burden of dementia and its changes in China, the United States, and Japan in 1990 and 2021** | | | | | | | |
| --- | --- | --- | --- | --- | --- | --- | --- |
| **Index** | | cases（UI） | | rates（UI） | | | **EAPC(95% CI)** |
|  |  | **1990** | **2021** | **1990** | **2021** | **percentage change** |  |
| **Prevalence** |  |  |  |  |  |  |  |
| China | Male | 1511602 (1280688 to 1737520) | 6162198 (5142286 to 7141800) | 574.55 (493.64 to 666.55) | 731.21 (618.54 to 851.63) | 0.27 (0.22 to 0.31) | 0.51 (0.44 to 0.57) |
|  | female | 2512934 (2165052 to 2892724) | 10828630 (9315735 to 12515957) | 785.19 (681.22 to 900.41) | 1025.11 (879.04 to 1186.81) | 0.31 (0.26 to 0.34) | 0.46 (0.36 to 0.55) |
| Japan | Male | 330505 (284410 to 379324) | 1066135 (916277 to 1229854) | 536.39 (463.45 to 613.48) | 551.02 (477.19 to 631.94) | 0.03 (0 to 0.05) | 0.3 (0.25 to 0.35) |
|  | female | 692722 (600657 to 789083) | 2301223 (1977426 to 2645284) | 718.87 (625.93 to 817.62) | 759.99 (657.37 to 866.96) | 0.06 (0.04 to 0.08) | 0.35 (0.29 to 0.42) |
| USA | Male | 902324 (778049 to 1035122) | 902324 (778049 to 1035122) | 730.61 (630.66 to 838.84) | 668.28 (570.17 to 775.3) | -0.09 (-0.11 to -0.07) | -0.31 (-0.36 to -0.25) |
|  | female | 1834030 (1582530 to 2102516) | 1834030 (1582530 to 2102516) | 856.88 (741.18 to 977.2) | 855.19 (742.39 to 975.4) | 0 (-0.02 to 0.01) | -0.02 (-0.07 to 0.02) |
| **Deaths** |  |  |  |  |  |  |  |
| China | Male | 39597 (9247 to 113675) | 163343 (40664 to 466660) | 25.12 (6 to 70.58) | 25.9 (6.51 to 73.2) | -0.01（0.03 to -0.04） | -0.03 (-0.07 to 0.01) |
|  | female | 80212 (19176 to 212442) | 328431 (83715 to 862460) | 34.61 (8.32 to 90.6) | 33.8 (8.6 to 87.19) | 0.00（0.04 to -0.03） | -0.2 (-0.24 to -0.17) |
| Japan | Male | 10079 (2480 to 27978) | 45200 (11589 to 118300) | 20.4 (5.02 to 55.61) | 20.35 (5.14 to 53.74) | 0.03（0.26 to -0.15） | 0.01 (-0.02 to 0.03) |
|  | female | 26893 (7004 to 68195) | 127607 (38480 to 282641) | 30.48 (8.14 to 76.04) | 29.57 (8.61 to 66.49) | -0.02（0.23 to -0.21） | -0.07 (-0.11 to -0.02) |
| USA | Male | 27343 (6708 to 74651) | 65527 (16822 to 172925) | 25.8 (6.57 to 68.6) | 25.6 (6.61 to 67.08) | 0.00（0.05 to -0.05） | -0.04 (-0.05 to -0.03) |
|  | female | 72614 (19149 to 183519) | 132519 (36475 to 321291) | 31.58 (8.29 to 80.01) | 31.6 (8.49 to 78.01) | -0.03（0.08 to -0.12） | -0.02 (-0.03 to 0) |
| **DALYs** |  |  |  |  |  |  |  |
| China | Male | 972799 (434085 to 2307848) | 3572279 (1694716 to 8148478) | 429.85 (186.23 to 998.45) | 463.67 (214.26 to 1055.84) | 0.08 (-0.07 to 0.27) | 0.09 (0.05 to 0.12) |
|  | female | 1729685 (790232 to 3750294) | 6500199 (3171765 to 13681029) | 596.71 (265.21 to 1288.3) | 631.38 (305.95 to 1318.24) | 0.06 (-0.13 to 0.26) | -0.02 (-0.06 to 0.02) |
| Japan | Male | 205150 (94878 to 462036) | 732872 (334666 to 1576628) | 356.71 (160.45 to 793.49) | 360.62 (165.48 to 774.81) | 0.01 (-0.02 to 0.04) | 0.1 (0.08 to 0.13) |
|  | female | 483410 (228443 to 1030432) | 1806422 (875587 to 3463156) | 517.87 (245.02 to 1092.53) | 516.08 (256.41 to 1001.7) | 0 (-0.09 to 0.07) | 0.06 (0.03 to 0.09) |
| USA | Male | 535598 (251040 to 1177657) | 1149557 (522331 to 2480337) | 456.02 (210.41 to 986.03) | 437.32 (197.33 to 940.67) | -0.04 (-0.07 to -0.02) | -0.16 (-0.18 to -0.14) |
|  | female | 1253458 (596545 to 2644339) | 2169086 (1032546 to 4481322) | 570.03 (270.84 to 1202.31) | 563.46 (270.18 to 1168.59) | -0.01 (-0.03 to 0.01) | -0.06 (-0.08 to -0.04) |
| EAPC:the estimated annual percentage change | | | | | | | |
